# Contrast arthrography of the equine temporomandibular joint

**DOI:** 10.3389/fvets.2024.1368131

**Published:** 2024-03-18

**Authors:** Clara S. Kim, Nathalie A. Reisbig, James L. Carmalt

**Affiliations:** Department of Large Animal Clinical Sciences, Western College of Veterinary Medicine, Saskatoon, SK, Canada

**Keywords:** horse, TMJ, radiography, imaging, intra-articular disc

## Abstract

**Background:**

Disorders of the equine temporomandibular joint (TMJ) cause clinical problems and detailed investigations of this joint are becoming more common. Specialist radiographic projections have the potential to highlight osseous abnormalities; however, the ability to assess the intra-articular soft tissue structures is currently limited to computed tomography (with, or without contrast enhancement) or magnetic resonance imaging. Both modalities are expensive and not readily accessible.

**Objective:**

To develop a technique of contrast arthrography of both compartments of the equine TMJ in cadavers and then perform the refined technique in three living horses as a proof-of-principle.

**Study design:**

A descriptive, experimental, study.

**Methods:**

Contrast arthrography of the discomandibular and discotemporal joint compartments of both TMJs was performed in 12 cadaveric equine heads using needles placed in the caudal pouches of the respective joint compartments. Radiographs were taken using previously published techniques, repeated with the mouth open and after air had been injected into the joints, to perform a double-contrast study. The TMJs of three healthy horses were subsequently examined to determine the validity of the procedure in live animals.

**Results:**

Single and double-contrast arthrography allowed delineation of the dorsal and ventral surfaces of the intra-articular disc in addition to filling the rostral and caudal joint pouches of the independent joint compartments. Contrast extravasation was common, and in two instances iatrogenic disc penetration resulted in the false diagnosis of pathologic disc perforation. The techniques were well tolerated in all three live horses.

**Main limitations:**

Low number of horses.

**Conclusion:**

Contrast arthrography allows interpretation of intra-articular soft tissue structures, but caution is advised in diagnosing intra-articular disc perforation. Even with experience, accessing the discomandibular joint can be challenging.

## 1 Introduction

The temporomandibular joint (TMJ) is comprised of two independent joint compartments separated by a biconcave fibrocartilagenous intra-articular disc. The discotemporal joint (DTJ) compartment lies above the disc and, in the horse, is composed of the articular tubercle, the mandibular fossa and retroarticular process of the temporal bone. The discomandibular joint (DMJ), located below the disc, contains the condylar process of the mandible and is the smaller of the two joint compartments. TMJ abnormalities occur commonly in humans and domestic animals. Intra- and extra-capsular conditions are recognized in humans, whereas in animals, the focus has been primarily on intra-capsular disease – specifically osteoarthritis. Advanced imaging forms an integral part of the diagnostic process when investigating human TMJ pain. Computed tomography (CT), either conventional or cone-beam, is considered the superior modality to examine osseous changes, whereas magnetic resonance imaging (MRI) is best for examining the intra-articular disc (1, 2). MRI generates static images, so contrast-enhanced arthrography using fluoroscopy is used for dynamic assessment of the joint to investigate possible disc adhesions or perforations (3, 4). Double-contrast enhancement of the human TMJ was developed in the 1960s and has since been superseded by MRI (5–7).

Diagnostic imaging of the equine TMJ has been reported to include radiography, ultrasound, CT and MRI (8–12). Specialized radiographic projections have been established to view the TMJ and limit the overlap of other cranial structures (13–15). Relative to the bone margins of the joint, the intra-articular disc is radiolucent (soft tissue radiodensity), unless there is dystrophic mineralization associated with the structure. Contrast CT has been used to assess the integrity of the joint compartments and the intra-articular disc, but this modality is not readily accessible to a significant portion of the horse-owning population (16). Single- and double-contrast arthrography has been used to examine the joints of the appendicular skeleton and may be beneficial in the investigation of the equine TMJ.

The objectives of this study were two-fold. Firstly, to develop a technique of radiographic contrast arthrography for both compartments of the equine TMJ in cadavers mimicking that already published using computed tomography, and secondly to perform the refined technique in three living horses as a proof-of-principle.

The hypotheses were that contrast arthrography of the equine TMJ would result in delineation of the intra-articular disc and detection of disc perforation (if present in the study population); and that the margins of the joint and extent of the synovial cavities would be readily appreciable using this technique.

## 2 Materials and methods

### 2.1 Phase I – cadaver specimens

The study population comprised of 12 cadaver heads with full adult dentition, which had been euthanized for reasons unrelated to the study and kept, with owner permission, for instructional purposes. The heads were placed on the edge of a steel table on their mandibles. The mouth of each head was opened maximally using a speculum (Stubbs Equine Innovations, Johnson City, TX) to counter the postmortem and freezing effects on the muscles of mastication. The jaw could subsequently be freely manipulated through its normal range of motion. The speculum was removed prior to any further intervention. The same investigator (JLC) performed all joint injections (cadaver and live horses) to ensure consistency.

**Figure 1:**
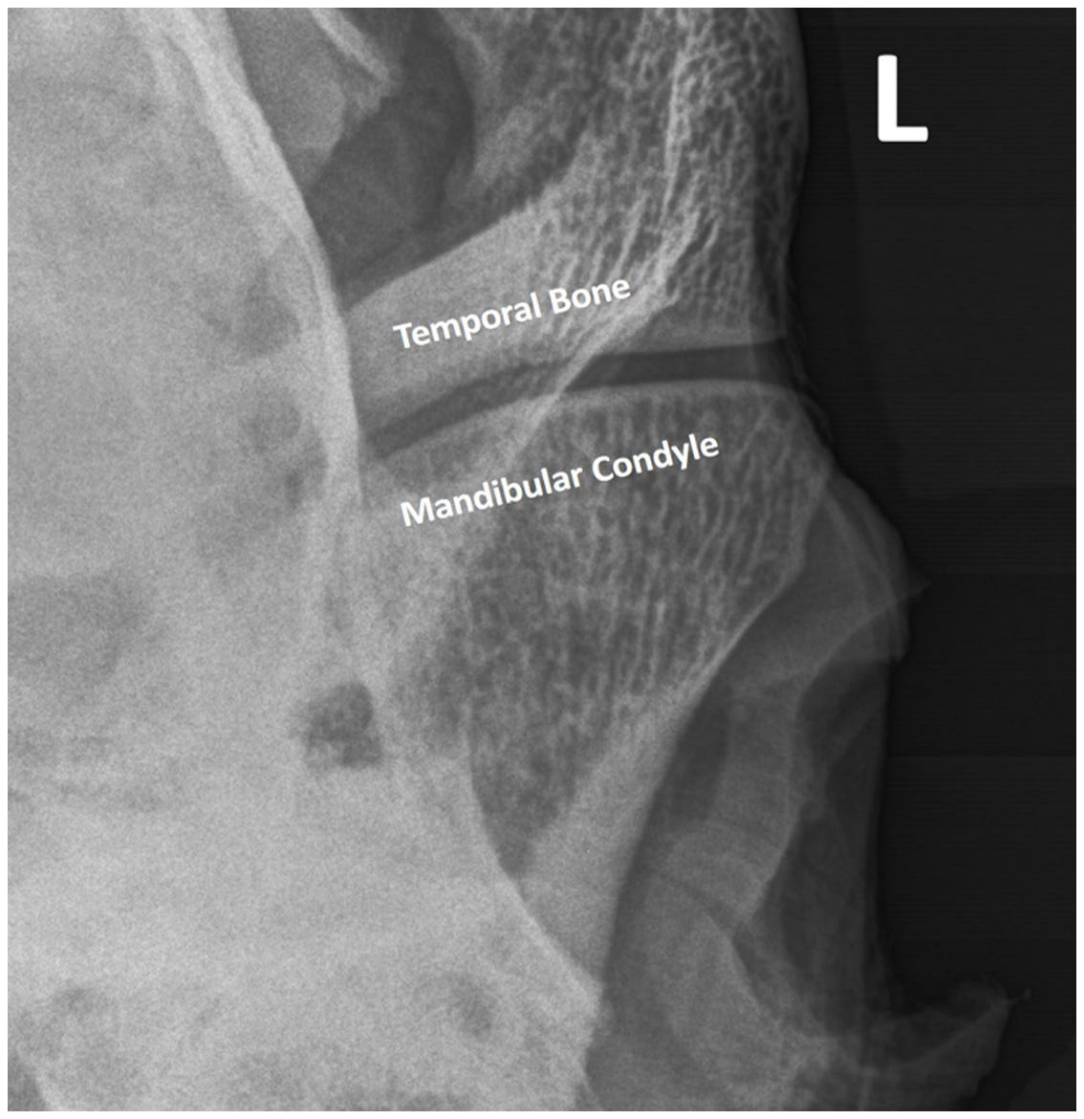
The radiographic image obtained using the R45°V30°L-CdDLO projection. The intra-articular disc is not delineated but is present between the temporal bone and mandibular condyle.

A previously reported closed-mouth radiographic projection (R45°V30°L-CdDLO) was used to obtain images of the left TMJ (Figure 1) (14), using digital radiography (Sound NEXT Equine DR, Sound, Carlsbad, CA, United States). This same projection was repeated with the horse’s mouth held open using a 2″ roll of duct tape inserted on its side. Then, the mouth was closed and a right lateral dorsal to left lateral ventral oblique (RtLD-LLVO) projection was taken to highlight the left TMJ. The opposite lateral oblique was used to delineate the right TMJ.

The mandibular condyle and intra-articular disc of the left TMJ were identified by palpation, and a 22G 1″ needle was inserted into the caudal recess of the DMJ in a lateral to medial direction, immediately dorsal to the condylar process of the mandible, under the intra-articular disc. 3 mL of 240mgI/mL iohexol (Omnipaque, GE Healthcare Canada, Inc., Mississauga, Ontario) was injected, and a lack of resistance to fluid flow was taken as an indication of successful entry into the joint. As contrast was injected into the joint, an appreciable filling of the ventrolateral recess of the joint was noted as previously reported (16). Immediately after injection, the mandible was manipulated through 5 complete masticatory cycles (the entire range of motion) before radiographs were repeated (mouth closed, mouth open and lateral oblique projections). Next, the DMJ was injected with 3 mL of air, the mandible was manipulated, and radiographs were repeated. The left DTJ was then injected with 3 mL contrast material using the lateral approach to the caudal pouch as described by Rosenstein et al. (17). Radiographs were taken as previously described, and for a final time after 3 mL air was injected to perform a double contrast arthrogram.

### 2.2 Phase II – live animals

**Figure 2:**
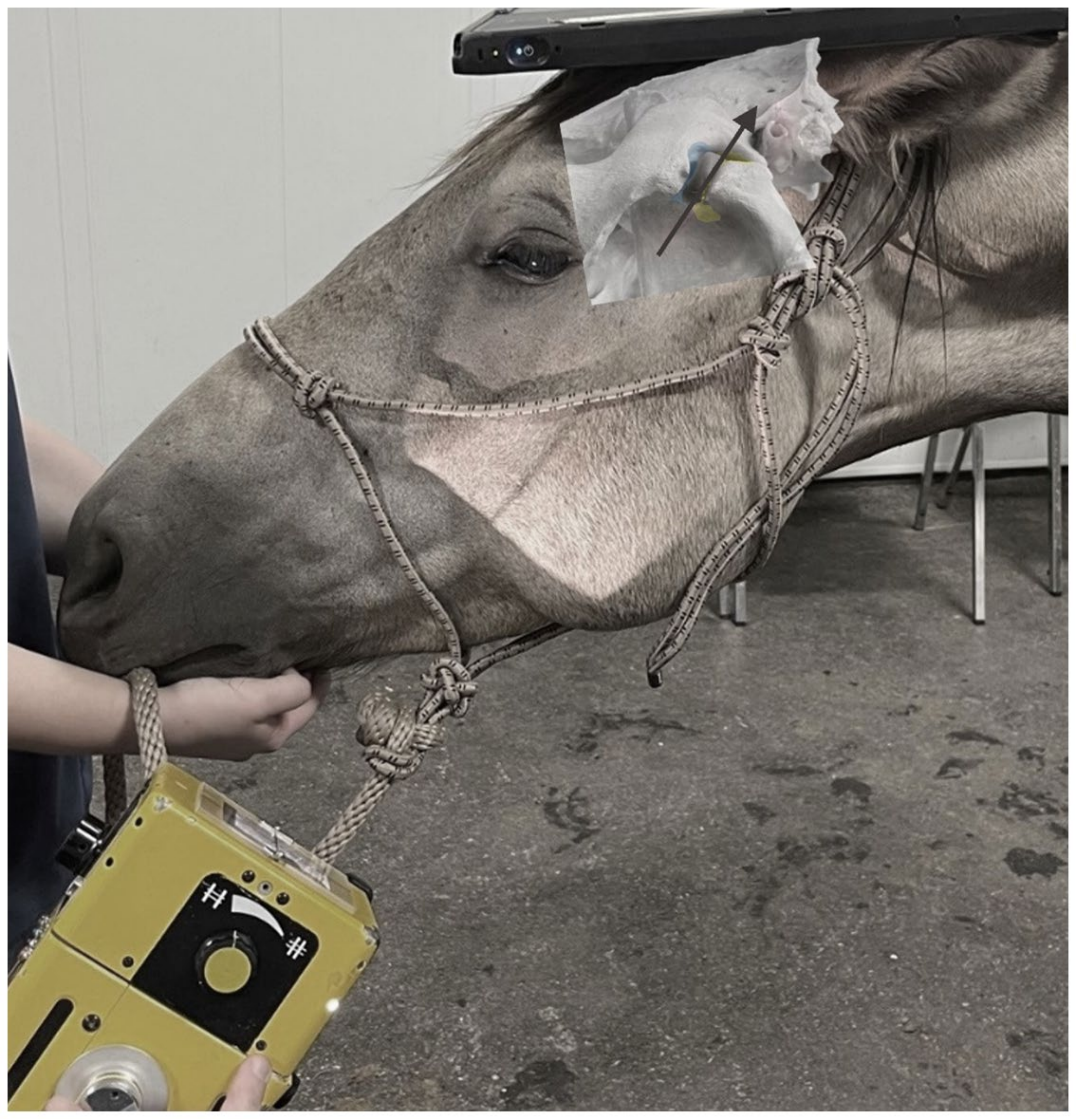
The generator and image capture device position while taking a radiograph of the TMJ using the R45°V30°L-CdDLO projection in a live horse. Beam angle is denoted by the arrow.

Three horses with suspected TMJ disease, donated for teaching purposes, were used in this portion of the study. Horses were chemically restrained using 2 mg detomidine hydrochloride and 2 mg butorphanol tartrate injected intravenously into the left jugular vein. The skin caudal to the eye and cranial to the ear was aseptically prepared in a routine manner for joint injections. Joint injection, mandibular manipulation, and the sequence of radiographs were performed exactly as had occurred in the cadavers (Figure 2). The joints were accessed using a 21G butterfly catheter. 2 mL lidocaine 2% was instilled into the joint, after which 3 mL 350 mg/mL iohexol mixed with 100 mg gentamicin in a 12 mL syringe was injected. Prior to connecting the syringe containing the contrast/antibiotic combination, 3 mL air was aspirated and, by adjusting the angle of the syringe, the fluid could be injected followed by the air without disconnecting it from the butterfly catheter extension tubing.

## 3 Results

### 3.1 Phase I – cadaver specimens

Both joint compartments could be successfully injected using published techniques. Opening the mouth did not appreciably change the published (closed mouth) R45°V30°L-CdDLO projection image. The initial study design randomized the order in which the joint compartments were injected. However, the rostral pouch of the DTJ filled with contrast following injection and mandibular manipulation. This consequently obscured the image of the condylar process of the mandible that is normally acquired using the R45°V30°L-CdDLO projection. This discovery necessitated injecting the DMJ first in all subsequent cases.

**Figure 3:**
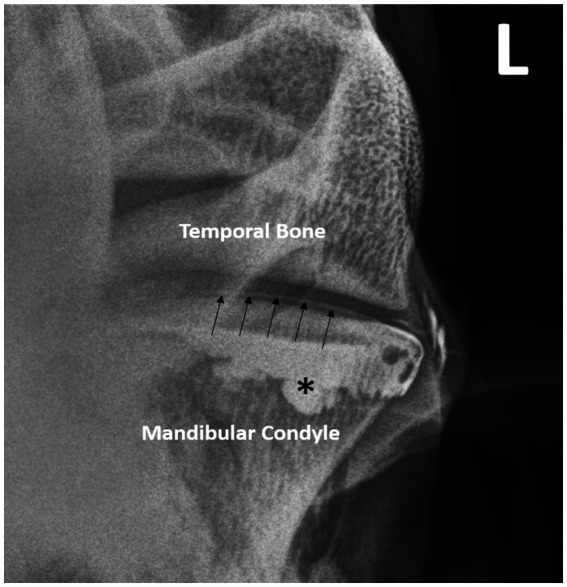
Double—contrast injection of the DMJ pools in the rostral pouch (*) and delineates the ventral border of the intra-articular disc (arrows). Note the contrast extravasation lateral to the joint.

Double contrast DMJ injection resulted in an appreciable filling of the rostral pouch of the joint compartment, as well as delineation of the ventral aspect of the intra-articular disc (Figure 3). Contrast extravasation was a common finding after joint injection, despite using a small gauge needle. Smaller gauge needles (25G) were trialed to reduce the incidence of extra-capsular contrast delivery, but the viscosity of the contrast agent prevented successful injection. There were two horses in which contrast material was detected in the DTJ after contrast agent and air were injected into the DMJ of the left TMJ. These joints were subjected to gross post-mortem examination after the conclusion of the study. The intra-articular discs were removed, and a small perforation was identified in each case. Given the size and location of the defects, the conclusion was that iatrogenic disc penetration had occurred during the delivery of the contrast agents.

**Figure 4:**
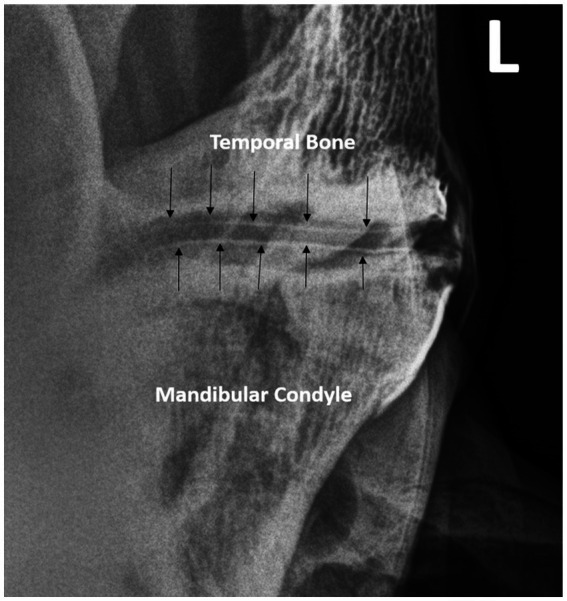
Double -contrast injection of both joints in a different horse delineates the dorsal and ventral margins of the intra-articular disc (arrows).

Contrast administration into both joint compartments allowed for the dorsal and ventral margins of the intra-articular disc to be imaged (Figure 4).

**Figure 5:**
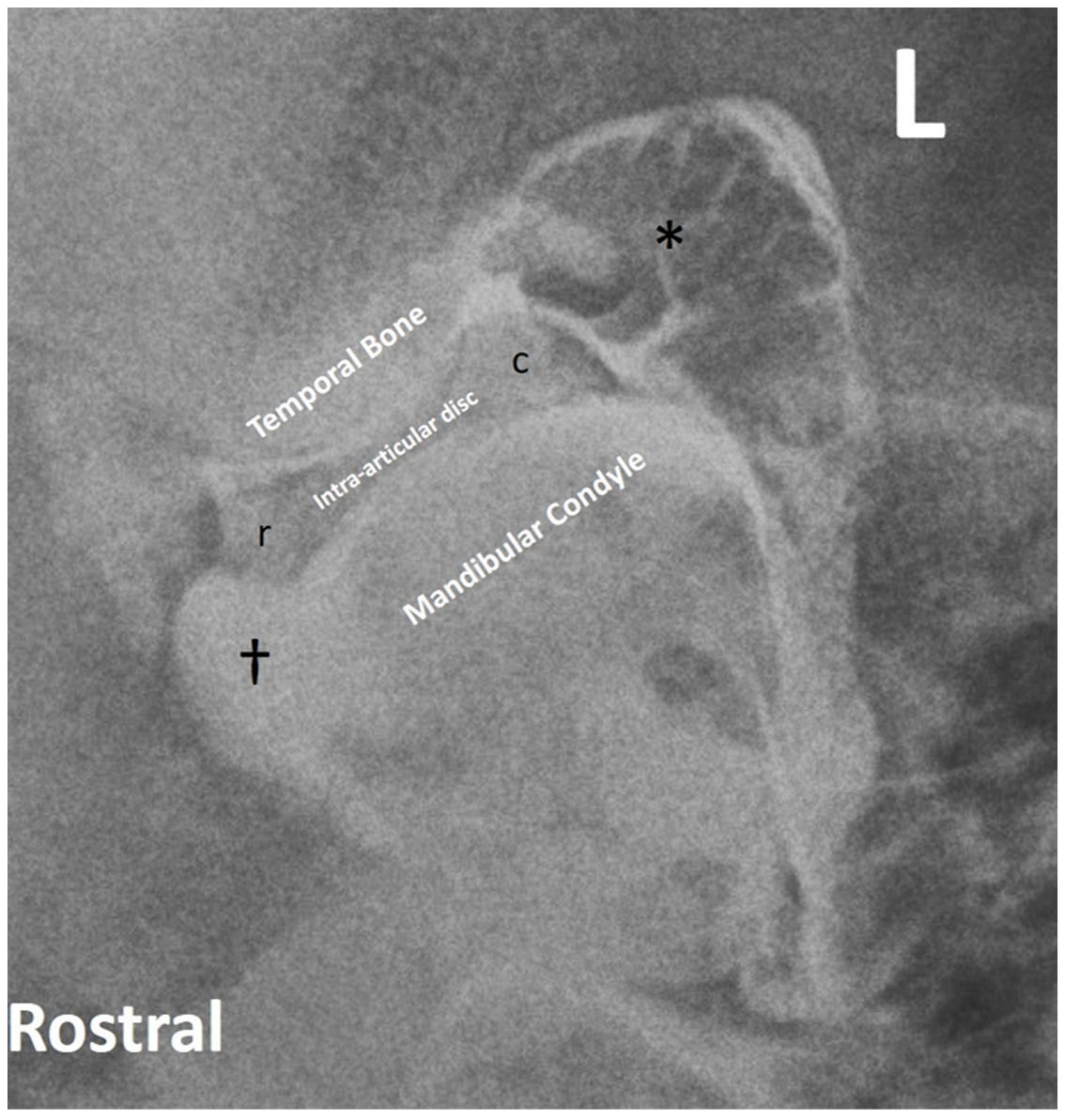
Double contrast lateral oblique radiographic projection of the left TMJ delineating joint margins (*), rostral (r) and caudal (c) disc, and contrast pooling (†).

Double-contrast (Figure 5) was superior to single-contrast arthrography for delineation of the joint margins. It illustrated the narrowing of the contrast columns as they flowed under and outlined the extremities of the intra-articular disc. Contrast pooling in the rostral and caudal pouches of the joint compartments was also evident.

### 3.2 Phase II – live animals

The images obtained in the live animals were comparable to those achieved in the cadaver specimens. Open mouth projections were not used for reasons outlined above. Extravasation of contrast agent was common and more than one attempt at needle placement was necessary in 50% of DMJs. Despite these complications, the horses were extremely tolerant of the intervention and stood for injection, mandibular manipulation, and imaging without additional restraint.

## 4 Discussion

The results of our study confirmed the hypotheses that the intra-articular disc could be successfully delineated, and the extent of the joint margins and synovial pouches could be readily determined. Indeed, the images obtained were similar to the computed tomographic images (axial projections) previously reported by the research group (16).

The rostral pouch of the DTJ is expansive and extends below (ventral to) the rostro-dorsal surface of the intra-articular disc. Therefore, when using the R45°V30°L-CdDLO projection, contrast agent overlies the dorsal aspect of the intra-articular disc and articular tubercle, obscuring them from view. The present study found that even though contrast material also pooled in the rostro-ventral pouch of the DMJ, the small volume used (3 mL) settled below the joint surface leaving a clear demarcation between the condylar process of the mandible and the ventral aspect of the intra-articular disc. These findings indicated that the DMJ should be subjected to contrast injection prior to evaluating the DTJ.

Another important finding was that the needle used to inject contrast agent could be inadvertently passed through the intra-articular disc. In both cases, this occurred while attempting to enter the DMJ and the error resulted in contrast material being present in both joint compartments simultaneously. This diagnostic finding mimics that associated with pathological tears of the disc and could potentially lead to erroneous diagnoses or further, unnecessary, diagnostic tests such as arthroscopy (18–20). The condylar process of the mandible slopes medioventrally away from the skin with the intra-articular disc in close apposition. Consequently, the 22G 1″ needle can impact the underside of the curving disc if it is directed straight into the DMJ, 90° to the skin surface. Possible solutions were to avoid deeper penetration into the joint, bend the needle to approximate the radius of curvature, or use a shorter needle. More care was subsequently used when inserting the 22G 1″ needles in the cadavers, and the 21G butterfly catheters in live horses, and no further anomalies were seen.

Discomandibular joint injection was challenging, despite the operator having extensive experience injecting both TMJ compartments. A recent report concluded that ultrasound guidance did not improve the accuracy of TMJ arthrocentesis in the horse (21). However, this paper only focused on the DTJ and it is possible that arthrocentesis of the DMJ is facilitated by using this modality. Alternatively, more practice or a different approach to the joint could improve the success rate of first time injection.

Overall, double contrast arthrography of the equine TMJ delivers more information than non-contrast techniques specifically pertaining to demarcation of the joint and the surfaces of the intra-articular disc. Despite these additional advantages, the resolution and multiplanar reconstruction techniques available with CT (especially when contrast enhanced), still make the latter technique preferable.

## 5 Conclusion

Single and double contrast arthrography of the equine TMJ allows delineation of the joint and intra-articular disc margins as well as determining the integrity of the disc. Inadvertent disc penetration during contrast administration may result in a false positive indication of disc compromise. Injection technique is not easy and must be performed carefully. Clinically, the clear presence of contrast agent in both joint compartments after single compartment injection is highly suggestive of an abnormality in the integrity of the intra-articular disc. That said, the absence of contrast in both compartments does not preclude pathology.

## Data availability statement

The original contributions presented in the study are included in the article/supplementary material, further inquiries can be directed to the corresponding author.

## Ethics statement

The animal study was approved by University of Saskatchewan Animal Research Ethics Board. The study was conducted in accordance with the local legislation and institutional requirements.

## Author contributions

CK: Investigation, Methodology, Writing – original draft, Writing – review & editing. NR: Conceptualization, Investigation, Methodology, Project administration, Supervision, Writing – original draft, Writing – review & editing. JC: Conceptualization, Investigation, Methodology, Supervision, Writing – original draft, Writing – review & editing.
